# The impact of PrsA over-expression on the *Bacillus subtilis* transcriptome during fed-batch fermentation of alpha-amylase production

**DOI:** 10.3389/fmicb.2022.909493

**Published:** 2022-08-04

**Authors:** Adrian S. Geissler, Line D. Poulsen, Nadezhda T. Doncheva, Christian Anthon, Stefan E. Seemann, Enrique González-Tortuero, Anne Breüner, Lars J. Jensen, Carsten Hjort, Jeppe Vinther, Jan Gorodkin

**Affiliations:** ^1^Department of Veterinary and Animal Sciences, Center for non-coding RNA in Technology and Health, University of Copenhagen, Copenhagen, Denmark; ^2^Department of Biology, University of Copenhagen, Copenhagen, Denmark; ^3^Novo Nordisk Foundation Center for Protein Research, University of Copenhagen, Copenhagen, Denmark; ^4^Novozymes A/S, Bagsværd, Denmark

**Keywords:** alpha-amylase, PrsA, *Bacillus subtilis*, RNA sequencing (RNA-seq), enzyme produced by microorganism

## Abstract

The production of the alpha-amylase (AMY) enzyme in *Bacillus subtilis* at a high rate leads to the accumulation of unfolded AMY, which causes secretion stress. The over-expression of the PrsA chaperone aids enzyme folding and reduces stress. To identify affected pathways and potential mechanisms involved in the reduced growth, we analyzed the transcriptomic differences during fed-batch fermentation between a PrsA over-expressing strain and control in a time-series RNA-seq experiment. We observe transcription in 542 unannotated regions, of which 234 had significant changes in expression levels between the samples. Moreover, 1,791 protein-coding sequences, 80 non-coding genes, and 20 riboswitches overlapping UTR regions of coding genes had significant changes in expression. We identified putatively regulated biological processes *via* gene-set over-representation analysis of the differentially expressed genes; overall, the analysis suggests that the PrsA over-expression affects ATP biosynthesis activity, amino acid metabolism, and cell wall stability. The investigation of the protein interaction network points to a potential impact on cell motility signaling. We discuss the impact of these highlighted mechanisms for reducing secretion stress or detrimental aspects of PrsA over-expression during AMY production.

## Introduction

*Bacillus subtilis* is a powerhouse for enzyme production in biotech industries (Schallmey et al., 2004; van Dijl and Hecker, 2013; Hohmann et al., 2016). Amylases are a specific class of enzymes that *B. subtilis* can produce commercially (Schallmey et al., 2004). The amylase enzyme, in particular the alpha-amylase (AMY), is a digestive enzyme (EC 3.2.1.1) that degrades starch molecules. Therefore, AMY is often an active component in laundry detergent for removing sticky stains from clothes. For successful AMY production and subsequent recovery, a host organism needs to both express and secrete AMY proteins in a biologically active form at a high rate (Spinnler, 2021). However, a major issue for commercial production is that the protein folding system of the cell is overwhelmed by the high rate of synthesis unless the strains used for production are genetically modified (Kontinen and Sarvas, 1993). The accumulation of unfolded AMY proteins causes stress that requires a bacterial cell to physiologically adapt to survive (Storz and Hengge, 2010). The Sec secretion pathway secretes AMY co-translationally (Fu et al., 2007). Therefore, unfolded AMY is extracellular, such that the corresponding stress signal triggers the heat shock response (Westers et al., 2004, 2006; Storz and Hengge, 2010; Lim and Gross, 2014; Yan and Wu, 2019). The simplified mechanism of this stress response has two components as follows (Westers et al., 2004, 2006; Storz and Hengge, 2010; Lim and Gross, 2014; Yan and Wu, 2019): First, the membrane-bound CssS receptor transduces the stress signal by phosphorylating CssR. Second, the phosphorylated CssR activates transcription of the two proteases, namely HrtA and HrtB, which degrade unfolded proteins and alleviate the stress condition. Furthermore, stress responses are intertwined with additional regulation in the core energy metabolism (Storz and Hengge, 2010), and such stress responses upregulate flagellar cell motility in order for a cell to physically escape the stress-causing location (Helmann et al., 1988; Márquez-Magaña et al., 1990; Yan and Wu, 2019). For instance, the level of cell motility is boosted by a low level of phosphorylated DegU (Kobayashi, 2007; Verhamme et al., 2007; Gupta and Rao, 2014), which is part of the core stress regulating DegU-DegS two-component system (Storz and Hengge, 2010; Laub, 2014). Nevertheless, these stress alleviating mechanisms can be opposed to the objective of achieving a high AMY yield: (i) the proteolytic degradation of AMY reduces yields, and (ii) a low phosphorylation level of DegU downregulates AMY expression (Gupta and Rao, 2014).

A state-of-the-art approach, which prevents the yield detrimental impact on the secretion of the stress response, is the over-expression of PrsA (Vitikainen et al., 2001; Quesada-Ganuza et al., 2019). Although the over-expression of PrsA reduces secretion stress by aiding AMY folding, it also has detrimental impacts such as hampered cell growth and even cell lysis (Vitikainen et al., 2001; Quesada-Ganuza et al., 2019). These detrimental phenotypes might be caused by protein-protein interactions of specific PrsA protein domains with still unknown partner proteins (Quesada-Ganuza et al., 2019). Another unknown aspect of PrsA over-expression is its impact on the bacterial transcriptome, particularly during industrial fed-batch fermentation. The adaptation to glucose metabolism from maltose metabolism has a global impact on half of all transcriptional regulators even though both carbons are preferred by *B. subtilis* (Buescher et al., 2012). Thus, we would assume a substantially larger global impact on the transcriptome for the extreme secretion stress during PrsA over-expression (Quesada-Ganuza et al., 2019). We consider our assumption to be further supported by a large number of over a hundred proteins that require regulation to adapt bacterial motility (see above concerning stress) (Rajagopala et al., 2007). Furthermore, a pure protein-coding gene focus ignores the essential role regulatory small RNA (sRNA), RNA chaperones, and non-coding RNA (ncRNA) have in facilitating physiological changes impacting the entire cell during stress responses (Storz and Hengge, 2010). General stress regulatory mechanisms have been investigated in public datasets (Arrieta-Ortiz et al., 2015); however, metabolic and stress pathways undergo complex temporal adaptations (Hahne et al., 2010; Otto et al., 2010). Thus, both temporally resolved and condition-specific gene expression levels are needed to study stress pathways. Specifically for secretion stress during *B. subtilis* AMY fed-batch fermentation, no such dataset exists to our knowledge.

Here, we conducted fed-batch fermentation of two commercial *B. subtilis* strains. Both strains produce an AMY and are isogenic, except that one of them over-expresses PrsA. We studied the transcriptome during fermentation at six timepoints with RNA-seq and analyzed the expression levels of both known coding and non-coding annotations, and also of potential novel transcribed, yet unannotated regions. We complemented the differential expression analysis with a network analysis of known protein–protein interactions (PPI). This study found significant changes in gene expression levels between the studied strains for genes in the ATP biosynthesis and cell motility biological processes. Furthermore, the network analysis hints at mechanisms relating to competence transformation and cell motility that might be candidates for further tuning of AMY secretion yields.

## Materials and Methods

### Strains and fed-batch fermentation

The overall experimental setup is as previously described in Geissler et al. (2022). In summary, *B. subtilis* strain 168 Δ*spoIIAC*Δ*amyE*Δ*apr*Δ*nprE*Δ*srfAC* was maintained at 4°C on the LBGG medium. The AMY JE1 [sequence label *je1zyn* in Geissler et al. (2022)] was inserted by splicing by overlapping extension (SOE, inserted sequences are in Supplementary Table 2) linear recombinant transformation, together with the commercial *sigA* promoter sequence P4199 and chloramphenicol marker in the *pel* locus. The PrsA over-expressing strain (referred to as the “+prsA” strain) had the insert by SOE of P4199, *prsA*, and spectinomycin marker in the *amyE* locus. A control strain without the prsA insert was included. After inoculation on SSB4 agar at 37°C, transfer on M-9 medium, sucrose 2M fed-batch fermentations were conducted in proprietary 2L tanks at 38°C. To avoid excessive overflow metabolite formation and to keep the culture in a sucrose metabolizing state, the fermentations were run as fed-batch fermentations without an initial batch phase. The feed medium consisted of sucrose (708 g/L), which was fed at a rate that increased linearly from 0.04 g/min at time = 0 h to 0.125 g/min at time = 8 h. The feed rate after 8 h of cultivation was kept constant at 0.125 g/min. Based on the dissolved oxygen tension data, the cultures entered a carbon-limited state after 9.4 h ± 0.53 of fermentation. Fermentations were run in triplicates for 5 days. The selected replicate size allows detecting significant logFC in the expression of at least ± 0.5 magnitude, as determined in benchmarks (Schurch et al., 2016). Samples were taken at six timepoints: 21, 26, 45, 71, 94, and 118 h after fermentation started. The samples were measured in cell density (OD650), and AMY activities were measured with an in-house assay. The assay (after 1/6,000 dilution) states the enzyme amount that breaks down 5.26 g starch per hour. This activity measure is proportional to the enzyme yield.

### RNA-sequencing dataset

All samples were immediately mixed with 5 ml of 100% ethanol and stored on dry ice. The RNA extraction and purification method is the identical phenol-chloroform protocol of Geissler et al. (2022). RNA libraries and sequencing were conducted by BGI Hong Kong with DNBseq in single-ends of 50 bp length. RNA libraries were prepared with 3′ adapter sequence AAGTCGGAGGCCAAGCGGTCTTAGGAAGACAA and the 5′ adapter AAGTCGGATCGTAGCCATGTCGTTCTGTGAGC CAAGGAGTTG. The 36 samples (triplicates, two strains, and six timepoints) were sequenced in three batches with technical replicates for QC (Supplementary Table 1). The computational analyses were conducted in an adapted workflow of Geissler et al. (2022) (doi: 10.5281/zenodo.4534403), which provides a pipeline in a Snakemake framework nested in computational reproducible Anaconda environments (Koster and Rahmann, 2012). In concordance with the read quality assessment of FastQC (version 0.11.8) (Andrews, 2010), any adapter contaminations were removed with Trimmomatic (version 0.39) for up to two seed mismatches at a minimal 10 bp sequence overlap and 30 bp palindromic overlap (Bolger et al., 2014). In a sliding window of 4 bp, reads were clipped for average Phred score quality below 20. From the 3′ of reads, positions with quality below 3 were removed. Finally, a minimal length of 40 bp was required for filtered and cleaned reads. Reads were mapped against the respective +prsA and control genome sequence with Segemehl (version 0.3.4, default settings) (Hoffmann et al., 2009). The mapping and QC filtering statistics are given in Supplementary Table 2. Expression levels of coding and non-coding annotations (see below) in the respective strains were quantified for uniquely mapping reads with featureCounts (subread version 1.6.4, ≥50% overlaps). Annotation coordinates in the respective strains were determined by liftOver (version 377) from the reference assembly (NC_000964.3) based on a pairwise alignment with LASTZ (version 1.0.4) (Harris, 2007; Liao et al., 2014; Haeussler et al., 2019).

### Novel potentially transcribed regions

Reference annotations of coding, non-coding RNA (ncRNA), transcripts, untranslated regions (UTRs), and RNA structures were used from the BSGatlas (version 1.1). The BSGatlas uses separate annotation entries to specify which regions of an mRNA transcript are the coding, untranslated, or potential *cis-*regulatory RNA structure parts. Such a distinguishment to the UTR element has advantages since *cis-*regulatory RNA structures can overlap coding regions. The BSGatlas unifies multiple databases and annotation resources, such that it also includes annotations for well-known ncRNA. Additional 141 putative ncRNA annotations from a tiling-array study were used (which are not part of the BSGatlas) (Nicolas et al., 2012; Geissler et al., 2021). Relative to these reference annotations and all transcript and untranslated regions (UTRs) annotated in the BSGatlas, we checked our RNA-seq data for transcription signals in 1,645 unannotated regions. The additional tiling-array annotations and un-annotated regions were determined with the R library plyranges (version 1.6.0) and GenomicRanges (version 1.38.0) combined with an overlap helper script from BSGatlas’ analysis code (doi: 10.5281/zenodo.4305872) in R (version 3.6.3) (R Development Core Team, 2008; Lawrence et al., 2013; Lee et al., 2018). Un-annotated regions shorter than 100 bp (the minimum length for >99% of the transcripts in the BSGatlas) were excluded from any further expression analysis. The expression counts for all coding/non-coding sequences and *cis*-regulatory RNA structures were normalized with DESeq2’s size-factor estimation (version 1.26.0) (Love et al., 2014). With respect to the downstream analysis of expression signals, we excluded the UTR annotations for improved interpretability, although we still retained all structured RNA *cis*-regulatory annotations. With the possible overlap between *cis*-regulatory RNAs and coding sequences, reads mapping within such overlaps can be counted twice during the quantification of expression. For a total of 542 unannotated regions, we observe expression signals of normalized read counts relative to gap length of at least 4/50 bp (corresponds to four times average coverage) (Supplementary Figure 1). We chose not to narrow down the transcribed regions because we found that a read coverage-based approach (as suggested in the workflow used in the RNA-seq dataset, last section) resulted in fragmented results (see example in Supplementary Figure 9). These regions were assumed to be *novel potentially transcribed regions* (NPTRs) (see Supplementary Table 3); all other unannotated regions were excluded from the subsequent expression analysis. We used the open reading frame (ORF) predictions of prodigal (version 2.6.3, default settings) to check for potential not-yet annotated coding elements within NPTRs (Hyatt et al., 2010). We also verified the overall quality of the ORF predictions by checking for overlaps with all known coding gene annotations of the BSGatlas. For each overlapping ORF-gene overlap (as detected by plyranges, see above), we computed the Jaccard similarity, which is the ratio of the length in the intersection of both annotations over their union.

### Differential expression analysis

**FIGURE 1:**
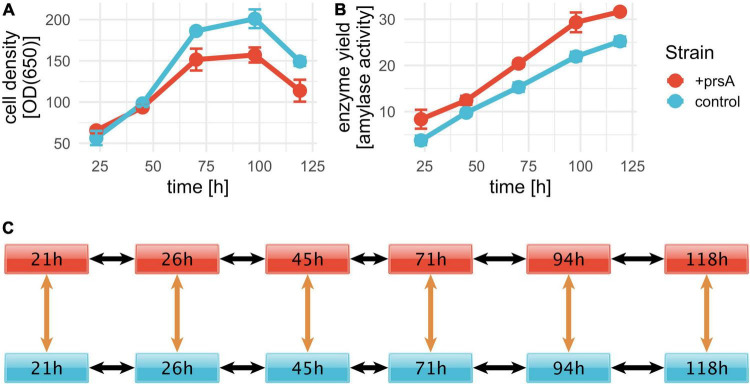
AMY fed-batch fermentations. Fed-batch fermentation was conducted in triplicates for a control strain (blue) and +prsA (red). RNA-seq samples were prepared at six timepoints: 21, 26, 45, 71, 94, and 118 h after fermentation start. Cell density and enzyme yield were measured for five timepoints: 23.2, 45, 70.2, 97.8, and 119 h. **(A)** The average cell density per strain over fermentation time was measured in optical density (OD) at 650 nm. The error bars indicate the standard deviation. **(B)** With a progressing fermentation, the yield increases. The shown yield is measured in enzyme activity (see section “Strains and fed-batch fermentation”). **(C)** For the differential expression, we investigated the significance of differential expression between the samples at six pairwise comparisons (orange arrows) and changes in expression over time in either strain for each pair (black arrows).

The expression levels of the coding/non-coding sequences, NPTRs, and *cis*-regulatory RNA structures were assessed for biological reproducibility in expression counts with scatter plots (Supplementary Figure 2). The scatter plots did not indicate visually striking patterns of batch effects according to the sequencing plan (Supplementary Table 1). The principal component analysis (PCA) inspection of the top 100 most variants that expressed annotations (without further diff. expression analysis) confirmed the relevance of the experimental design in the latent structure of the expression data with the principal components corresponding to the strains and time aspect (Supplementary Figure 3). Differential expressions for pairwise comparisons between the strains at each of the six time points and within each strain along the time axis (Figure 1C) were assessed with the DESeq2’s Wald test. Similar to the analysis presented in Geissler et al. (2022), the pairwise tests were weighted in a stage-wise procedure to guarantee an overall false discovery rate relative to the number of annotations: each annotation was screened for dynamic expression with a log-ratio test against a static expression model before confirming which of the pairwise tests had changes in expression. The screening and pairwise tests included a linear factor in the regression models to account for potential batch effects. The stage-wise weighting was conducted with stageR (version 1.8.0) (Van den Berge et al., 2017) and differential expression was called for adjusted *p*-values < 0.01. Overall, 2,127 annotations were detected as differentially expressed (Table 1 and Supplementary Table 4). Based on the z-scaled log expected mean expression levels (Supplementary Table 5), expression profiles were grouped in 10 k-means clusters (R implementation). The profiles per strain were clustered separately (one gene = two rows in the data matrix). The number of clusters was determined by the “elbow method” over the total within-cluster error curve (Supplementary Figure 4; Thorndike, 1953).

**TABLE 1:** Differentially expressed annotations.

Annotations	No. Annotations considered for analysis	Differentially expressed	Strain-specific expression
CDS	2,674	1,791 (67.0%)	1,026 (57.3%)
NPTRs	355	234 (65.9%)	123 (52.6%)
Putative ncRNA	107	68 (63.6%)	38 (55.9%)
Riboswitch	37	20 (54.1%)	10 (50.0%)
tRNA	22	9 (40.9%)	7 (77.8%)
sRNA	9	3 (33.3%)	2 (66.7%)
Synthetic PrsA	1	1 (100.0%)	1 (100.0%)
Synthetic AMY	1	1 (100.0%)	0
asRNA	1	0	0
SRP	1	0	0

For the differential expression analysis, multiple coding and non-coding annotations were considered (first column). The number of genes with minimal expression levels as determined by DESeq2’s independent filtering, which were inspected for potential differential expression, is in the second column. The number of detected differentially expressed annotations in any of the pairwise comparisons (Figure 1C) is in the third column. The last column lists the number of annotations detected to have a significant difference in expression between the strains.

The percentages provided in parenthesis are relative to the columns to the left (Only 355 of the 542 NPTRs passed the independent filtering).

### Regulated biological processes

We investigated the set of differentially expressed genes and their upregulation and downregulation for over-representation in biological processes as annotated in Gene Ontology (GO) terms, which are readily available for 78.3% of coding genes (Caspi et al., 2014; Geissler et al., 2021). For each pairwise differential expression test (Figure 1C), we inspected the set of upregulated genes (those with a positive logFC) and downregulated genes separately. The over-representation analysis was performed with topGO (version 2.37.0) (Alexa and Rahnenführer, 2022). Over-representation for the respective upregulated and downregulated genes was determined with a fisher test for the significance level of 0.01 relative to the background of all expressed genes, which were determined by the DESeq2’s independent filtering procedure. This procedure discards the on average lowly expressed genes in order to maximize the number of differentially expressed genes (indicated by NA for *p*-values in Supplementary Table 4; Love et al., 2014). The minimal term size was set to 10, and the dependencies due to GO’s hierarchy were de-correlated with topGO’s “elim” algorithm. After filtering for a minimal observed/expected ratio of magnitude 2 (between the 80 and 85th percentile), *p*-values were adjusted for multiple testing with a false discovery rate (FDR). The over-represented processes and the associated differentially expressed genes are listed in Supplementary Tables 6, 7 and Supplementary Figure 6.

### Protein–protein interaction network analysis

The protein–protein interaction network analysis was conducted in Cytoscape (version 3.8.2) (Shannon, 2003) for the differentially expressed protein-coding genes (both with and without significant logFC between strains). High-confidence protein associations (confidence score >0.8) were retrieved from the STRING v11 database using stringApp (version 1.6.0) for the *B. subtilis* strain *168* (Doncheva et al., 2019; Szklarczyk et al., 2019). The resulting network was clustered with the MCL algorithm (inflation value of 2.5, confidence scores as edge weights) implemented in the clusterMaker2 app (version 1.3.1) (Enright, 2002; Morris et al., 2011). The visualization of significant between strain logFCs on the network nodes was added with Omics Visualizer (version 1.3.0) (Legeay et al., 2020).

### Global amino acid composition

In order to interpret the regulated biological processes (see above), we inspected the global amino acid compositions of all *B. subtilis* protein-coding genes. The nucleotide sequences of all coding sequences from the BSGatlas were extracted with BSgenome (version 1.54.0) (Pagès, 2021). The corresponding amino acid sequences were determined according to the bacterial genetic code with Biostrings (version 2.54.0) (Pagès et al., 2019). Here, we used only the 99.3% of the coding genes that were completely relative to their corresponding amino acid sequences; that is, they used all codons encoded in their nucleotide sequences, correctly started with methionine, and ended with a stop codon. The composition in average proportion was determined for these complete sequences (Table 2).

**TABLE 2:** Amino acid composition.

Amino acid	All coding genes	Diff. expressed	Highly expressed	AMY	PrsA
**Tryptophan**	1.03% ± 0.99	1.06% ± 0.96	0.86% ± 0.81	**4.12% (+3.1 SD)**	0.35% (−0.7 SD)
**Asparagine**	4.07% ± 2.05	3.87% ± 1.75	3.86% ± 1.44	**8.82% (+2.3 SD)**	2.82% (−0.6 SD)
Histidine	2.30% ± 1.51	2.27% ± 1.33	2.12% ± 1.10	4.31% (+1.3 SD)	1.06% (−0.8 SD)
Tyrosine	3.57% ± 1.95	3.39% ± 1.56	3.13% ± 1.32	5.49% (+1.0 D)	3.17% (−0.2 SD)
Glycine	6.67% ± 2.74	6.95% ± 2.41	7.62% ± 2.08	8.82% (+0.8 SD)	6.34% (−0.1 SD)
**Aspartic acid**	5.11% ± 2.29	5.06% ± 2.10	5.17% ± 1.83	6.86% (+0.8 SD)	**11.27% (+2.7 SD)**
Threonine	5.32% ± 1.87	5.44% ± 1.75	5.51% ± 1.35	5.88% (+0.3 SD)	4.23% (−0.6 SD)
Arginine	4.19% ± 2.14	4.07% ± 1.91	4.24% ± 1.92	4.31% (+0.1 SD)	0.70% (−1.6 SD)
Proline	3.48% ± 1.71	3.55% ± 1.49	3.93% ± 1.24	3.53% (+0.0 SD)	0.35% (−1.8 SD)
Glutamine	3.87% ± 2.04	3.85% ± 1.82	3.65% ± 1.42	3.73% (−0.1 SD)	6.34% (+1.2 SD)
Alanine	7.36% ± 2.84	7.82% ± 2.65	8.25% ± 2.29	7.06% (−0.1 SD)	7.39% (+0.0 SD)
Phenylalanine	4.63% ± 2.40	4.53% ± 2.20	4.21% ± 1.94	4.31% (−0.1 SD)	1.76% (−1.2 SD)
Valine	6.75% ± 2.37	6.92% ± 2.11	7.38% ± 1.91	6.27% (−0.2 SD)	6.69% (−0.0 SD)
Methionine	2.46% ± 1.43	2.52% ± 1.31	2.50% ± 1.11	2.16% (−0.2 SD)	1.76% (−0.5 SD)
Serine	6.22% ± 2.26	6.28% ± 2.16	6.04% ± 2.04	4.51% (−0.8 SD)	4.93% (−0.6 SD)
Cysteine	0.91% ± 1.13	0.84% ± 0.91	0.70% ± 0.72	0.00% (−0.8 SD)	0.35% (−0.5 SD)
**Lysine**	7.50% ± 3.14	7.13% ± 2.78	6.79% ± 2.17	4.90% (−0.8 SD)	**17.96% (+3.3 SD)**
Glutamic acid	7.37% ± 3.29	7.20% ± 3.13	7.23% ± 2.77	4.31% (−0.9 SD)	9.15% (+0.5 SD)
Leucine	9.70% ± 3.06	9.72% ± 2.86	9.42% ± 2.35	6.67% (−1.0 SD)	8.45% (−0.4 SD)
Isoleucine	7.50% ± 2.65	7.53% ± 2.48	7.37% ± 2.15	3.92% (−1.4 SD)	4.93% (−1.0 SD)

The average amino acid compositions (rows) are shown for all B. subtilis endogenous (thus excl. AMY and PrsA) coding genes (second column), those that were detected as differentially expressed (third column), and the by average expression 10% most highly expressed genes (fourth column). The standard deviations are shown behind the “±” signs. The compositions of amino acids for the AMY enzyme (fifth) and the over-expressed PrsA (sixth) column are shown. The difference in standard deviations relative to the average for all genes are indicated in parenthesis. The bold font highlights amino acids with difference of more than two standard deviations.

## Results

### Novel potentially transcribed regions

#### Transcriptome analysis from RNA-seq data

To elucidate potential mechanisms of *B. subtilis* secretion stress during the production of the AMY enzyme JE1 (commercial name Natalase™) with a particular focus on PrsA over-expression, we conducted fed-batch fermentation in triplicates for two isogenic strain conditions: one control strain and one strain with PrsA over-expression (from here on referred to as +prsA). As expected from the reduced growth upon PrsA over-expression (Vitikainen et al., 2001; Quesada-Ganuza et al., 2019), the +prsA strain has a lower cell density (Figure 1A) and higher AMY yield (Figure 1B). To capture the transcriptome dynamics during fermentation, we took out samples for RNA-seq analysis at six timepoints: 21, 26, 45, 71, 94, and 118 h after fermentation started. These timepoints correspond to sampling every 24 h (within a 3 h window) with one additional sample at the early phase of the fermentation.

#### Transcriptional activity for the reference annotations

In order to comprehensively investigate both the coding and non-coding RNA elements, we quantified the RNA-seq expression according to a recently developed transcript atlas for *B. subtilis* (Geissler et al., 2021). We included 141 additional annotations from a tiling-array study that was not included in the atlas due to unclear mechanism of transcription (annotations were ambiguous as to whether they are independent full RNA transcripts or only part thereof) (Nicolas et al., 2012; Geissler et al., 2021). In the following, we refer to these annotations, together with the less well-characterized RNA elements from the atlas, as putative ncRNA. These reference annotations combine gold standard curated information, computational RNA structure biology, and transcriptomic analysis of over 100 experimental conditions (Nicolas et al., 2012; Geissler et al., 2021). Additionally, these experimental conditions suggest that still 5% of remaining unannotated regions have evidence of expression activity (Geissler et al., 2021). Fed-batch fermentations were not part of the above-mentioned experimental conditions, such that there might be a larger potential to discover fed-batch-related regions from our RNA-seq data. Consequently, we investigated our RNA-seq data for expression in such unannotated regions.

#### Novel potentially transcribed regions

There are a total of 1,645 un-annotated contiguous stretches of the genome or gaps (stranded, meaning there can be antisense located annotations) between reference annotations of length >100 bp (minimal length for 99.5% of transcripts in the atlas). We detect novel potentially transcribed regions (NPTRs) by inspecting the average RNA-seq read coverages over the entire unannotated gap region (read counts, DESeq2 size-factor normalized, relative to the lengths). Relative to the 50 bp sequencing lengths (see section “RNA-sequencing dataset”), 70% of atlas annotations were on average expressed by four reads and 30% by one read. In contrast, only 20% (542) of unannotated regions were on average covered by four reads. This high coverage for these 542 NPTRs (Supplementary Figure 1) indicates that the NPTRs may have functional importance and that it would be relevant to include these in subsequent expression analysis (see Supplementary Table 3).

### PrsA over-expression changes gene expression regulation of the global transcriptome

#### Differential expression

We assessed the impact of PrsA over-expression on the bacterial transcriptome by analyzing the expression levels of coding and non-coding sequences (see section “Transcriptional activity for the reference annotations” above), including the 141 additional annotations and the 542 NPTRs with DESeq2. For each region, we performed 16 pairwise differential expression tests: six tests between the two strains on each timepoint and 2 × 5 tests from one timepoint to the next in both strains (Figure 1C). Since each pairwise test corresponds to a separate hypothesis test, we used stage-wise testing to adjust for the overall false discovery rate (FDR) per annotation (Love et al., 2014; Van den Berge et al., 2017). Compared to controlling the FDR per hypothesis, the overall FDR increases statistical power and guarantees the FDR relative to the gene/annotation number, independent of the number of hypotheses (Van den Berge et al., 2017). As part of the differential expression analysis, DESeq2’s independent filtering detected about half of all coding sequences and 355 of 542 NPTRs as expressed (Love et al., 2014). At an overall FDR *p*-adj. ≤0.01, we detected differential expression for 1,793 coding sequences (67% of expressed genes), 234 NPTRs (66%), 68 putative ncRNAs (64%), 20 riboswitches (54%), 9 tRNAs (41%), and 3 sRNAs (33%) (Table 1 and Supplementary Table 4). The differentially expressed coding genes include the AMY enzyme and the over-expressed PrsA. Between 50 and 78% of these biotypes had strain-specific expression patterns (significant difference for at least one of the six between strain tests). PrsA had strain-specific expression (as expected by not being inserted into the control strain’s genome). Notably, no strain-specific expression was detected for AMY.

To further assess the coding potential of the differentially expressed NPTRs, we leveraged a set of 4,226 ORF predictions (Hyatt et al., 2010). These ORF recall 4,085 of 4,332 known coding sequences perfectly (overlap with Jaccard similarity >90%), which corresponds to a recall of 94.3% with a precision of 96.7%. Only 141 ORF predictions do not recall coding genes. Furthermore, only 18 overlap unannotated regions (>100 bp); for 3 ORF, the overlap is less than 5% of the ORF length (Table 3). Also, only two of the ORFs are fully located within an NPTR that has detected differential expression; in both cases, the ORFs antisense overlaps the 3′ ends of the coding genes: The electron transfer flavoprotein *etfA* and the gene of unknown function *yobB*.

**TABLE 3:** ORF overlapping un-annotated regions.

Overlapping gap	ID	Gap length	ORF start	ORF end	ORF strand	ORF length	% ORF within gap
Low expression	gap-865	18994	164501	164923	−	18994	100
Low expression	gap-964	382	635149	635262	−	382	100
Low expression	gap-1109	277	1233396	1233599	−	277	100
Low expression	gap-1244	15761	1784706	1784837	−	15761	100
Low expression	gap-1402	1357	2747665	2747853	−	1357	100
Low expression	gap-643	8279	3341033	3341254	+	8279	100
NPTR^†^	gap-1322	7336	2221838	2221951	−	7336	100
**NPTR with diff. expr.**	**gap-1293**	**743**	**2050930**	**2051037**	−	**743**	**100**
**NPTR with diff. expr.**	**gap-538**	**7687**	**2915229**	**2915390**	+	**7687**	**100**
Low expression	gap-1495	1272	3419414	3419656	−	1272	96.7
Low expression	gap-1367	140	2560095	2560259	−	140	84.8
NPTR^†^	gap-113	1795	718436	718597	+	1795	83.3
Low expression	gap-1166	197	1453322	1453525	−	197	79.4
**NPTR with diff. expr.**	**gap-1354**	**1038**	**2460278**	**2460625**	−	**1038**	**26.7**
NPTR without diff. expr.	gap-1296	581	2056650	2057114	−	581	23.2
**NPTR with diff. expr.**	gap-1058	2975	1033458	1034003	−	2975	4.8
Low expression	gap-449	2395	2555468	2555845	+	2395	2.1
Low expression	gap-90	2286	608879	609391	+	2286	1.4

Shown are each overlap between ORFs (that did not recall a coding gene) and un-annotated regions (>100 bp). Some of these un-annotated regions with sufficient expression signal were considered as NPTR and further processed for differential expression analysis (column 1–3). Bold texts highlights gaps with detected differential expression. Based on the coordinates of the predicted ORF (column 4–7), the overlap of the ORF with the un-annotated gap is expresses relative to the length of the ORF (column 8).

^†^Un-annotated region with sufficient expression signal to be considered as NPTR, but which was excluded from further analysis by the independent filtering procedure of the differential expression analysis.

#### The regions with the highest expression changes

The strain-specific expression patterns of PrsA and the respective logFC between the two strains on all six timepoints were the most extreme observed in this study with logFC values up to a factor of 20 at each timepoint. Other extreme logFC values were observed for genes from operons encoding a variety of biological functions (Table 4). The NAD biosynthesis genes of the *nadABC* operon (Rodionov et al., 2008) also have extreme logFC, but they undergo both extreme upregulation and downregulation in the control strain with *nadA* and *nadB* being downregulated from timepoint 21 to 26 h (both logFCs<−6, adj. *p* < 0.004) and subsequently upregulated from 26 to 45 h (both logFCs ∼+7, adj. *p* < 3e-10). Due to the secretion stress, the production strains attempt to sporulate despite being unable to do so (Geissler et al., 2022). Consistently, the two sporulation genes, namely *safA* and *coxA*, were among the most extremely regulated (logFC > 6, adj. *p* < 2.3e-5). Other extreme changes in expression (logFC < −5) were observed for the spore killing factors *skfA* and *skfB* (González-Pastor, 2011), the sporulation controlling factor *spoIIGA* (Ramos-Silva et al., 2019), the bacitracin resistance genes *bceA* and *bceB* (Ohki et al., 2003), the for NADH during fermentation essential lactate dehydrogenase *ldh* (Cruz Ramos et al., 2000; Larsson et al., 2005), and an NPTR antisense to the gene of unknown function *ytta* (Asai et al., 2007).

**TABLE 4:** Most extreme observed logFCs.

Name	Type	Test	logFC	Adj. *P*	Location
nadB	Coding	Between strains, 26 h	7.4	1.07E-10	2847871<-2849466
nadB	Coding	Control, 26->45 h	7.2	2.65E-10	2847871<-2849466
nadC	Coding	Control, 26->45 h	7.0	2.89E-09	2847048<-2847917
nadC	Coding	Between strains, 26 h	6.9	5.26E-09	2847048<-2847917
nadA	Coding	Control, 26->45 h	6.8	7.82E-11	2845955<-2847061
nadA	Coding	Between strains, 26 h	6.8	1.33E-10	2845955<-2847061
safA	Coding	Control, 26->45 h	6.4	4.05E-09	2844675<-2845838
safA	Coding	Between strains, 26 h	6.2	8.98E-09	2844675<-2845838
coxA	Coding	Between strains, 26 h	6.2	1.82E-05	2843931<-2844527
coxA	Coding	Control, 26->45 h	6.1	2.28E-05	2843931<-2844527
spoIIGA	Coding	Control, 26->45 h	−5.2	8.47E-67	1603779->1604708
skfB	Coding	Control, 26->45 h	−5.4	2.86E-60	214175->215407
skfA	Coding	+prsA, 26->45 h	−5.4	7.31E-09	213941->214108
skfA	Coding	Control, 26->45 h	−5.7	4.39E-24	213941->214108
bceA	Coding	Control, 26->45 h	−6.2	5.76E-107	3111327<-3112088
nadA	Coding	Control, 21->26 h	−6.2	0.002258927	2845955<-2847061
bceB	Coding	Control, 26->45 h	−6.5	1.62E-127	3109397<-3111337
nadB	Coding	Control, 21->26 h	−6.5	0.003566971	2847871<-2849466
ldh	Coding	+prsA, 21->26 h	−6.6	2.42E-13	329774->330739
gap-1449	NPTR	control, 26->45 h	−6.9	1.85E-48	3108525<-3109352

The table lists the top 10 most extreme upregulated and downregulated genomic elements according to their logFC of differential expression (fourth column). prsA is excluded since it was upregulated with an approximate logFC of 20 between strains at all-time points. For each genomic element, the locations (last column) are relative to the reference genome (see section “Materials and methods”). The pairwise tests (third column) refer to the conducted differential expression analysis (Figure 1C), and the corresponding adjusted P-values are listed in the fifth column.

#### Biological processes and differentially expressed genes are mutually associated

**FIGURE 2:**
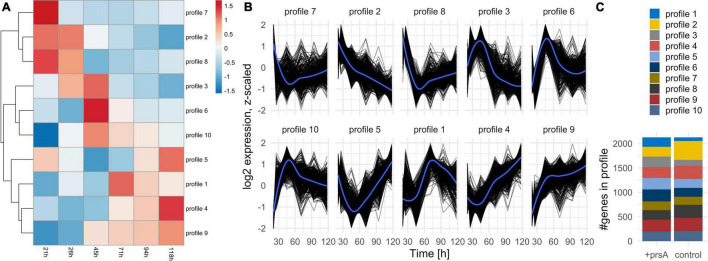
Expression profiles. **(A)** Heatmap of the expression profile over time (columns) for all differentially expressed coding and non-coding annotations investigated separately per strain. The resulting profiles were clustered (rows) and re-arranged by a complete linkage tree. **(B)** Profiles of expression per cluster for each annotation (black lines). An overall average expression according to a loess regression is added in blue. **(C)** The number of annotations per profile in either strain. The expression dynamics for each annotation can be in two separate profiles in the strains.

The investigation of the overall expression profiles from a k-means clustering (Figure 2A) on the average expected expression at each timepoint (Supplementary Table 5) shows marked differences in the expression dynamics between the strains (Figure 2C). Also, all profiles indicate a substantial shift in dynamics between timepoints 45–71 h, during which the cell population increased the most (Figure 1A): For instance, profiles 4 and 5 drop in expression levels at that timepoint but recover and even exceed the starting expression level whereas profiles 7 and 8 have drastically downregulated expression at that timepoint and do not recover (Figure 2B). Genes and other biotypes with strain-specific expression patterns had predominately different expression profiles between the strains, whereas those without strain-specific expression had the same (Supplementary Figure 5). Therefore, *B. subtilis* regulates gene expression both timepoint- and strain-specifically.

**FIGURE 3:**
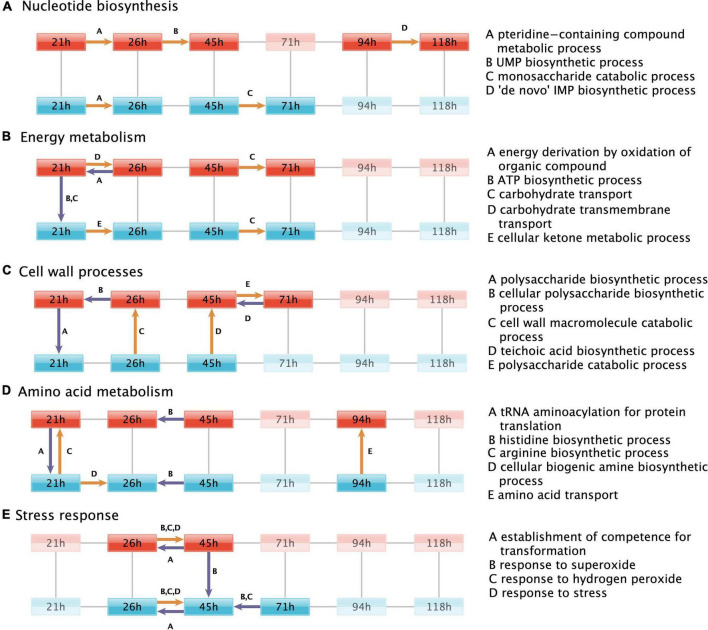
Regulated biological processes. Biological processes that are over-represented by the genes differentially expressed in each of the pairwise comparisons (black lines) between the fermentation timepoint in the +prsA (red) and control strain (blue). For simplicity, the regulated processes are grouped in subplots according to the same biological functions discussed in the result sections, which touch upon **(A)** nucleotide biosynthesis, **(B)** energy metabolism, **(C)** cell wall processes, **(D)** amino acid metabolism, and **(E)** stress response. Supplementary Figure 7 shows the regulated processes without further functional subdivision. Colored arrows indicate a pairwise comparison that was over-represented in a process (see description to the right). The arrows point to the conditions in which expression levels were higher. Upregulation in the +prsA strain or upregulation with time progression of the fermentation is highlighted in orange, whereas downregulation is shown in purple. In each subplot, time-strain conditions not adjacent to an arrow are grayed out.

We assessed which biological processes [annotated in Gene Ontology (GO), terms (Ashburner et al., 2000)] are over-represented among the differentially expressed genes in each time and strain pairwise comparison (Figure 1C). We compared the numbers of respective upregulated or downregulated genes relative to the number of expressed genes (see section “Materials and methods”). A total of 24 processes had significant over-representation (Fisher’s exact test, FDR *p*-adj. ≤0.01). We inspected the list of differentially expressed genes per process (Supplementary Table 7) in combination with meta-information available in the BSGatlas, particularly KEGG pathway annotations (Kanehisa and Goto, 2000; Geissler et al., 2021). Notably, the detected over-represented processes annotate genes with differentially expressed logFC predominately above the background logFC distribution of genes without detected differential expression (Supplementary Figure 8). Furthermore, some of the top 10 most extremely upregulated and downregulated genes (Table 4) were annotated by the detected processes (Supplementary Table 7), namely cell wall macromolecule catabolic process (*safA* and *skfA*), response to stress (*nadC* and *nadE*), and ATP biosynthetic process (*ldh*). We further inspected the detected biological processes (Figure 3) for their relevance with respect to fed-batch fermentation, as described in the sections later.

#### Nucleotide biosynthesis

It is well established that an ample supply of nucleotides is needed for efficient AMY protein expression (Hosoda et al., 1959) and thus also the nucleotide precursors, such as UMP and IMP, are of regulatory interest (Peifer et al., 2012; Hohmann et al., 2016). Consistently, the over-representation investigation indicates an upregulation of UMP (GO:0006222) and IMP (GO:0006189) biosynthesis in the +prsA strain from timepoint 26 to 45 h and 95 to 118 h, respectively. The monosaccharide catabolic genes (GO:0046365), especially the genes involved in the ribose synthesis *via* the pentose phosphate pathway (Supplementary Table 7), are upregulated in the control strain from timepoint 45 to 71 h. The pteridine-containing compound metabolic process (GO:0042558) was over-represented by genes upregulated from the first to the second timepoint in both strains. These specific genes are also part of the folate biosynthesis pathway, which is essential for both purine and pyrimidine synthesis (Kilstrup et al., 2005) and therefore quintessential for AMY production (Hohmann et al., 2016; Hosseini et al., 2018).

### PrsA over-expression affects genes involved in energy metabolism

#### ATP biosynthesis

The ATP biosynthetic process (GO:0006754) was significantly downregulated in +prsA compared to the control strain on the first timepoint of the fermentation. Furthermore, the data suggest that the energy derivation by oxidation of organic compounds (GO:0015980) was further downregulated in +prsA from the first to the second timepoint within the first day of fermentation. The differentially expressed genes associated with both processes comprise a long list (>50, see Supplementary Table 7) of core energy metabolic enzymes from the citrate cycle, oxidative phosphorylation, and glycolysis. Nevertheless, the list also overlaps with the starch and sucrose metabolism pathway, particularly with glycogen biosynthesis (*glgA, glgB, glgC, glgD*, and *glgP*) (Kiel et al., 1994). Consistent with these observations, the carbohydrate transport (GO:0008643) was also downregulated in +prsA on the first timepoint. In contrast, the cellular ketone metabolic process (GO:0042180) was upregulated in the control strain from the first to the second timepoint. Ketones are essential for the biosynthesis of menaquinone (Lu et al., 2008). Menaquinone is *B. subtilis’* respiration coenzyme, similar in function to ubiquinone in human mitochondria (Lemma et al., 1990). Nevertheless, the ATP biosynthetic process (GO:0006754) was not detected significantly over-represented by the regulated genes at the other fermentation timepoints.

#### Altering carbohydrate transport during fermentation

The over-representation analysis also suggests that both strains have an upregulated carbohydrate transport (GO:0008643, GO:0034219) from 45 to 71 h. The transport might also be upregulated in the +prsA strain from the first to the second timepoint.

### PrsA over-expression affects genes involved in cell wall destabilizing processes

Low PrsA protein abundances and increased concentrations of teichoic acid can reduce cell growth and cell wall disruption (Driessen et al., 1998; Hyyryläinen et al., 2000). For instance, the inhibition of the dlt operon–which is key to teichoic acid synthesis–increases AMY yields (Hyyryläinen et al., 2000; Yan and Wu, 2017). However, our data suggest that not only *dltB* expression is upregulated in +prsA on timepoint 45 h (logFC = 0.86, adj. *p* < 2.11e-5) but also the entire teichoic acid biosynthetic process (GO:0019350). Additional processes relating to cell wall molecules and polysaccharide biosynthetic (GO:0033692, GO:0000271) were observed as downregulated in +prsA. Nevertheless, not only does our data suggest that the biosynthesis is downregulated, the corresponding catabolic processes (GO:0016998, GO:0000272) might be upregulated.

### Upregulation of amino acid metabolism during PrsA over-expression

#### Regulated amino acid metabolism

Genes of the arginine biosynthetic process (GO:0006526) are over-represented among the genes upregulated in the +prsA strain on the first timepoint and for the amino acid transport (GO:0006865) at timepoint 94 h after fermentation started. The histidine biosynthetic process (GO:0000105) was detected as downregulated from timepoint 26 h to the timepoint 45 h in both strains. The data suggest also that the tRNA aminoacylation for protein translation (GO:0006418) is downregulated in +prsA on the first timepoint, and that the cellular biogenic amine biosynthetic process (GO:0042401) is upregulated in the control strain from the first to the second timepoint.

#### Expected changes in amino acid metabolism

Given the observed potential regulation in amino acid metabolism above, we investigated to which extent these might be the result of the peptide sequence of the secreted AMY. The inspection of the codon composition of all coding genes suggests that the AMY and the over-expressed PrsA contain substantially more tryptophan, asparagine, aspartic acid, and lysine (more than 2 standard deviations from the average proportion, Table 2). Tryptophan was the strongest over-represented amino acid in AMY (+3.1 standard deviations). In comparison, the subset of neither differentially expressed endogenous (excl. AMY and PrsA) coding genes nor 10% of most highly expressed endogenous genes have changes in the overall composition (within 1 standard deviation). The PrsA was extremely over-expressed in the +prsA strain (logFC > 19, adj. *p* ≤ 5.27e-40). By average expression, AMY was the 5th and PrsA the 34th highest expressed gene (see Supplementary Table 6). Thus, the enrichment of these four amino acids in AMY and PrsA should have physiological relevance: given the high energetic cost of tryptophan biosynthesis (Akashi and Gojobori, 2002), the evolutionarily adapted amino acid metabolism will be affected (Smith and Chapman, 2010).

### Protein–protein interactions of stress response and competence transformation

#### Stress response turning point

The over-representation investigation reveals that both strains upregulate parts of their stress response concerning the reactive oxygen species (ROS) response (GO:0006950 and the two children terms GO:0042542, GO:0000303) from timepoint 26 h to 45 h. Simultaneously, the strains downregulate the establishment of competence for transformation (GO:0030420). The protein ClpC is the key switch between heat shock (including secretion stress) and competence regulation (Turgay et al., 1997). During stress, a three-protein complex of ClpC, MecA, and ComK is formed (Turgay et al., 1997). The bound central competence regulator ComK can no longer act as a transcription regulator, which prevents the establishment of competence (Turgay et al., 1997). According to our results, *clpC* undergoes significant differential expression during fermentation in both strains, but neither *comK* nor *mecA* had significant expression changes though both were expressed (Supplementary Table 4). Given that the molecular mechanism of the ClpC switch (i) is post-translational, (ii) does not directly impact the transcription levels of the involved genes, and (iii) involves a third factor, the analysis by pairwise comparison of expression levels cannot detect that specific interaction. Therefore, we complemented the expression analysis with protein-protein interaction (PPI) network analysis.

#### Protein–protein interaction network analysis

**FIGURE 4:**
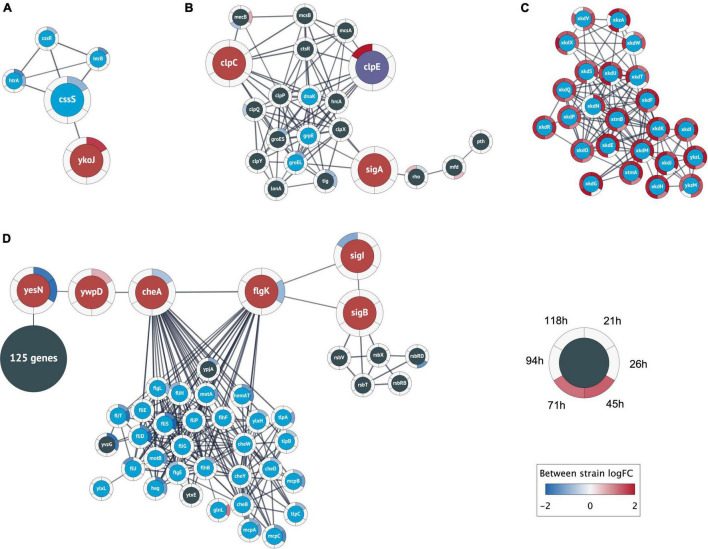
Relevant clusters of differentially expressed genes. Nodes represent protein coding genes and edges correspond to high-confidence protein interactions retrieved from STRING. The differential expression between strains is shown as rings around the nodes, where each ring contains the logFC values for each time point comparison in a blue-white-red color gradient (see figure legend). A high positive logFC is colored red and indicates a significantly larger expression in the +prsA strain compared to the control. Non-significant differential expression is shown as 0 logFC (white). The logFC color gradient was truncated at ±2. **(A)** The genes in this cluster include the central heat shock stress two-component system of CssRS and the proteases HtrAB (blue nodes). The cluster also contains the gene ykoJ of unknown function (red node) connected to the stress transducer CssS (large blue node). **(B)** This cluster contains the competence/heat shock switch protein ClpC (leftmost red node) and the universal sigma factor SigA (rightmost red node); SigA and ClpC share interactions with the tree heat shock proteins dnaK, grpE, and groEL (blue nodes). The cluster also contains ClpE (purple node) that had substantially higher expression in +prsA at timepoint 118 h (logFC ∼2.6). **(C)** The analysis found a cluster of 24 prophage or prophage-like genes that were closely interacting and had significantly higher expression in +prsA throughout the fermentation. **(D)** The largest cluster contains a “bottleneck” of high-confidence interactions at two genes of unknown function (yesN and ywqD) between 125 genes of various catalytic function (summarized as one node) and 29 chemotaxis genes (blue nodes) and the central chemotaxis signal protein CheA, the flagellar hook-filament FlgK, the general stress repose sigma factor SigB, and the RNA polymerase sigma factor SigI.

We retrieved PPIs from the STRING database for the *B. subtilis* strain 168. STRING provides a list of functional associations from multiple evidence channels, such as curated knowledge from known metabolic pathways and protein complexes, physical PPIs from lab experiments (e.g., pull-down assays), predicted interactions from text mining of the biomedical literature, or associations based on co-expression analysis (Szklarczyk et al., 2019). The resulting network of 4,774 high-confidence associations (confidence score >0.8) among 1,770 of the 1,791 differentially expressed protein-coding sequences was clustered into 201 protein clusters using MCL (Enright, 2002; Morris et al., 2011; Doncheva et al., 2019). In combination with the significant logFCs between the +prsA and control strains (Legeay et al., 2020), we manually inspected four clusters with interesting patterns regarding this study’s outset (Figure 4). These are described in the following sections below.

#### Two-component system

The first PPI cluster consists of the CssRS two-component system, including the involved proteases (see Introduction, Figure 4A). However, the cluster contains an additional association between the stress signal transducer CssS and YkoJ of unknown function. The *ykoJ* expression during secretion of a vaccine compound (beta-toxoid) positively depends on CssS (Nijland et al., 2007). In contrast, the expression during AMY might have a negative dependency with *cssS* being significantly lower expressed in +prsA on timepoint 21 h after fermentation start (logFC = −0.9, adjusted *p* = 8.5e-10) and *ykoJ* significantly higher (logFC = 1.7, adj. *p* = 1.2e-7). To our knowledge, the association YkoJ-CssS has not been characterized in the context of AMY production.

#### Competence switch

The second cluster (Figure 4B) contains the above-described heat shock/competence protein switch ClpC (Turgay et al., 1997). The cluster also contains ClpC’ repressor CtsR (Derré et al., 1999) and the universal sigma factor SigA. Furthermore, SigA and ClpC share associations with the three heat shock proteins DnaK, RrpE, and GroEL. Although *mecA* was not detected as differentially expressed, the paralog *mecB* was, and it is part of this second cluster (Persuh et al., 2002). *B. subtilis’* other two Clp-proteins ClpP and ClpE are also part of this cluster. ClpE had a significantly higher expression on timepoint 118 h in +prsA (logFC = 2.6, adj. *p* = 0.0005), which is relevant because ClpE destabilizes the functionality of the repressor CtsR (Miethke et al., 2006).

#### Prophage genes

A third cluster (Figure 4C) contains a set of tightly associated 24 PBSX prophage and prophage-like genes that were all significantly higher expressed in +prsA compared to control at various timepoints during the entire duration of the fermentation (logFC ∈ [0.9, 2.4], adj. *p* ∈ [2.6e-23, 9.2e-3]). PBSX, a defective *B. subtilis* prophage (Wood et al., 1990a), is known to be potentially heat-induced (Wood et al., 1990b), and they have a potential association with the level of lytic stress resistance (Buxton, 1980).

#### Potential cell motility regulation

Finally, the fourth cluster has an interesting pattern of associations involving many chemotaxis genes (Figure 4D). This cluster is structured into two separate interconnected components: On the one side, there are 29 chemotaxis proteins and on the other 125 protein-coding genes with various catalytic functions [116 of 125 (92.8%) genes are annotated in the general catalytic activity term GO:0003824]; however, both parts are connected by a backbone of associated genes. This backbone includes the central flagella motion frequency regulator CheA, the flagellar hook-filament FlgK, the general stress sigma factor SigB, the heat-shock protein sigma factor SigI, and the two partially characterized signal transducers YesN and YwspD (Fabret et al., 1999; Petersohn et al., 2001; Zuber et al., 2001; Asai et al., 2007; Mukherjee and Kearns, 2014). Interacting with SigB are five stress regulatory proteins induced by SigB (according to STRING annotations). Both YesN and YwsqD are described as histidine kinases, although the corresponding response regulator remains unknown (Fabret et al., 1999; Caspi et al., 2014; Zhu and Stülke, 2018; Geissler et al., 2021). Even if the regulators are unknown, the backbone has an interesting pattern of antagonistic logFC: (i) YesN is significantly lower expressed in +prsA on timepoint 21 h (logFC = −1.7, adj. *p* = 1.4e-6) and 26 h (logFC = −1.84, adj. *p* = 8.5e-5), (ii) YwspD is higher expressed in +prsA on 21 h (logFC = 0.6, adj. *p* = 0.0037), and (iii) CheA lower again on 21 h (logFC = −0.7, adj. *p* = 0.0025). The bottom-line is that the PPI analysis elucidates the tight associations between heat shock, competence transformation, cell motility, general stress response, and translation.

## Discussion

In this study, we investigated how PrsA over-expression in *B. subtilis* impacts the transcriptome during fed-batch alpha-amylase (AMY) fermentation. We carried out a temporally resolved RNA-seq study to analyze expression levels and regulation of biological processes with respect to secretion stress. We inspected a comprehensive set of coding and non-coding reference annotations and 542 novel potentially transcribed regions (NPTRs). The fermentation process strongly affects gene expression and we observe a large number of differentially expressed genes both between the strange and overtime: a total of 1,793 coding genes (67% of expressed genes), 234 NPTRs (66%), 68 putative ncRNAs (64%), 20 riboswitches (54%), 9 tRNAs (41%), and 3 sRNAs (33%) were differentially expressed. The PrsA over-expressing strain, which is consistent with prior descriptions had increased yield and reduced growth (Quesada-Ganuza et al., 2019), was observed to have a significant strain-specific differential expression for more than half of the transcribed genes. Subsequent in-depth analysis of regulated biological processes (Figure 3) and the PPI network of differentially expressed coding genes (Figure 4) shed light on the complex intertwined processes of stress pathways, core energy metabolism, and cell motility (Helmann et al., 1988; Márquez-Magaña et al., 1990; Storz and Hengge, 2010; Yan and Wu, 2019).

Concerning the NPTR, we assessed their potential to contain ORF relative to predictions that recalled 94.3% of known genes with high precision of 96.7%. A marginal fraction of these ORF overlap unannotated regions (Table 3). Therefore, our data do not suggest the presence of ORF in the NPTR, including those with detected differential expression in this dataset. Future investigation for potential conservation of RNA—let alone assessment of their biological function—requires RNA structure alignments that can have average sequence identities below 40% (Yao et al., 2006, 2007; Weinberg et al., 2010). We predicted the NPTR relative to a reference transcript annotation that integrates a comprehensive set of annotation databases and resources (Nicolas et al., 2012; Caspi et al., 2014; Geissler et al., 2021; Pedreira et al., 2022). Among these resources is SubtiWiki, an active community effort that comprehensively collects previously identified coding and non-coding genes (Zhu and Stülke, 2018). Therefore, we consider the NPTR to extend beyond known transcribed regions.

### Amino acid and energy metabolism

The observation of the potentially downregulated ATP biosynthesis in the +prsA strain surprised us: (i) The AMY hypersecretion is stressful and energy-intensive for the cells (Song et al., 2015). (ii) It has been hypothesized that ATP might be required for PrsA chaperone activity (Yan and Wu, 2017). (iii) The reduction of ATP levels can also increase the general stress response of *B. subtilis* (Haldenwang, 1995; Petersohn et al., 2001; Yan and Wu, 2019). The potential downregulation of ATP biosynthesis in the +prsA strain seems counterintuitive because the strain has both lower stress and higher yield than the control (Quesada-Ganuza et al., 2019). However, the reduced ATP biosynthesis might be due to the impact of the hypersecreted AMY and over-expressed PrsA on amino acid metabolism. Contrary to the evolutionary energetic adaption of the amino acid composition for secreted proteins (Smith and Chapman, 2010), the four amino acids tryptophan, asparagine, aspartic acid, and lysine are over-represented in the AMY and PrsA proteins (Table 2). Although the specific metabolism processes for these four amino acids were not detected as significantly regulated during fermentation (Figure 3), more general amino acid processes (e.g., transport) or biosynthetic processes for other amino acids (arginine and histidine) were significantly over-represented by regulated genes. On the one hand, the upregulation of arginine synthesis and related transport mechanisms improves osmotic stress resistance (Du et al., 2011; Zaprasis et al., 2015), which in turn is beneficial to AMY production in *B. subtilis* (Zhao et al., 2018). On the other hand, the over-represented amino acids might explain the reduced ATP biosynthesis. (i) Tryptophan is the amino acid with the highest biosynthetic cost in *B. subtilis*, with a 42.9% higher cost than the second most costly amino acid (phenylalanine) (Akashi and Gojobori, 2002). (ii) The biosynthesis, in particular for costly amino acids, diverges intermediate metabolites from ATP biosynthesis (Akashi and Gojobori, 2002). In the case of tryptophan, the intermediate metabolites have already diverged from glycolysis, which also impacts the downstream citrate cycle (Kanehisa and Goto, 2000; Akashi and Gojobori, 2002). However, a more definite inspection to confirm the regulation of the amino acids and ATP metabolism would require an investigation of concentrations of the individual metabolites with for instance metabolomics.

### Cell wall destabilizing processes

The over-expression of PrsA is known to lead to reduced cell growth and cell lysis (Quesada-Ganuza et al., 2019). It was suggested that protein-protein interactions of specific PrsA protein domains are causal for these phenotypes (Quesada-Ganuza et al., 2019). Our data suggest that, on a transcription regulatory level, the PrsA-over-expressing stain has both increased polysaccharide catabolism and reduced polysaccharide biosynthesis. We hypothesize that this strongly contributes to cell wall breakdown, which leads to detrimental phenotypes. Therefore, investigating the associated differentially expressed genes could potentially be the outset to trace back the causality chain of why their regulation changes, and as a path forward to finding candidates that stabilize cell walls and increase yields. Furthermore, the PPI network analysis highlighted 24 tightly associated PBSX prophage and prophage-like genes (Figure 4C) that might be decisive in unraveling the PrsA over-expression lysis phenomena (Buxton, 1980; Quesada-Ganuza et al., 2019), particularly due to the heat-induced (and thus secretion stress-related) expression of the PBSX genes (Wood et al., 1990b).

### Stress and cell motility

The protein-protein interaction network analysis resulted in four clusters of proteins that we found to be relevant to this study’s outset (Figure 4). These were the genes of the CssRS two-component secretion stress response in one cluster (Figure 4A), while the known ClpC regulatory switch and its associations with secretion stress, competence transformation, and associations with the universal sigma factor SigA belong to another cluster (Figure 3B; Turgay et al., 1997). Furthermore, the analysis provided a large cluster (Figure 4D) of cell motility-related genes, which is consistent with the large number of proteins involved in regulating bacterial motility (Rajagopala et al., 2007). A closer inspection of the latter cluster suggests that the proteins YesN and YwsqD might have a signaling role in balancing between cell motility and 125 genes that are annotated to have various metabolic catalytic functions, e.g., the phosphogluconate dehydrogenase. To our knowledge, the potential relationship between cell motility and AMY fermentation has not been elucidated so far, although a potential hypothesis could be that the signaling facilitates the regulation of flagellar cell motility to escape from the stress region (Helmann et al., 1988; Márquez-Magaña et al., 1990; Yan and Wu, 2019). Nevertheless, a follow-up study is needed to verify cell motility regulation during AMY production.

### Conclusion

In conclusion, our transcriptome study highlights the expression dynamics of secretion stress during fed-batch AMY fermentation. The comparison of expression levels in a PrsA over-expressing strain to a control strain showed differential expression for nearly half of the transcribed genes. A wide variety of upregulated and downregulated biological processes is related to energy and amino acid metabolism. Also, the data shows potential associations of the cell lysis phenomenon of PrsA over-expression with the stress response and cell motility. Overall, these results identify genes and biological processes, which are affected during fermentation and by the overexpression of PrsA and provide a starting point for future genetic modification of *B. subtilis* for improved yield.

## Data availability statement

The genomic sequences and RNA-seq data were deposited in the GEO database (GSE189556). The expression coverages are presented as a browser for interactive investigation (http://rth.dk/resources/bsg/prsa). The annotations of the BSGatlas are accessible at https://rth.dk/resources/bsgatlas/. The additional putative ncRNA annotations are part of the supplementary information of Nicolas et al. (2012). The RNA-seq data were processed with a reproducible pipeline located at doi: 10.5281/zenodo.4534403.

## Author contributions

AG conducted the entire computational analysis and wrote the manuscript. LP extracted the RNA. ND contributed to the analysis and methodology design of the PPI network. CA contributed to the discussion of the expression analysis. EG-T contributed to the writing in the early stage. AB prepared the bacterial strains. LJ contributed to the discussion of gene clustering, enrichment analysis, and PPI network analysis. SES, CH, JV, and JG supervised the work. JG and AG made the study design. JG was the main project coordinator. All authors read and approved the manuscript.
